# Changing places to study the medium-term effects of air pollution: carotid arterial stiffness

**DOI:** 10.1186/2049-3258-73-S1-P21

**Published:** 2015-09-17

**Authors:** Hans Scheers, Lidia Casas, Tim Nawrot, Ben Nemery

**Affiliations:** 1 KU Leuven, Leuven, Belgium; 2 UHasselt, Hasselt, Belgium

## Background and aim

A biomarker for cardiovascular disease, carotid arterial stiffness is linked with exposure to air pollution. In a panel study on short- to medium-term health effects of air pollution, we evaluated the association between NO_2_ exposure and indicators of carotid arterial stiffness.

## Methods

Arterial stiffness was measured in 20 healthy volunteers (59-76 years of age) in three locations and at 11 timepoints during one year: seven times in Leuven (Belgium) and twice during each 10-day stay in Milan (Italy) and Vindeln (Sweden). Pulse Wave Velocity (PWV), distensibility (DC) and compliance (CC) were measured using Esaote MyLabOne ultrasound. Personal NO_2_ exposure, an indicator of traffic-related air pollution, was monitored during 5 consecutive days before each health assessment using passive samplers. Associations between arterial stiffness and exposure to NO_2_ were evaluated with linear mixed models, adjusting for sex, age, heart rate, arterial pressure, and time.

## Results

In Milan, NO_2_ was higher by about 40 μg/m^3^ and in Vindeln it was lower by about 15 μg/m^3^ than in Belgium. A 10 μg/m^2^ increase in NO_2_ was associated with an average increase in PWV of 0.087 m/s (95% confidence interval (CI): 0.016-0.157 m/s). Adjusting for personal and weather characteristics did not alter the result (+0.104 m/s; CI: 0.002-0.206). A 10 μg/m^3^ increase in NO_2_ was also associated with a decrease in DC (adjusted coefficient: -0.039 kPa^-1^; CI -0.061 - -0.017) and CC (-0.031 mm^2^/kPa; CI -0.055 - -0.008). Similar results were obtained when using PM_10_ (obtained from monitor stations, the average of lag0 to lag6) as the exposure variable.

## Conclusion

Given that increased PWV and decreased DC and CC indicate greater arterial stiffness, we found in a real life intervention study that exposing healthy elderly to higher or lower air pollution results in concurrent changes in carotid arterial stiffness.

